# An online tool for information to women with epilepsy and therapeutic drug monitoring in pregnancy: Design and pilot study

**DOI:** 10.1002/epi4.12473

**Published:** 2021-02-20

**Authors:** Kristina Lisovska, Eva Gustafsson, Judith Klecki, Anna Edelvik Tranberg, Johan Zelano

**Affiliations:** ^1^ Department of Neurology Sahlgrenska University Hospital Gothenburg Sweden; ^2^ Department of Clinical Neuroscience Institute of Neuroscience and Physiology Sahlgrenska Academy Gothenburg University Gothenburg Sweden; ^3^ Wallenberg Center of Molecular and Translational Medicine Gothenburg University Gothenburg Sweden

**Keywords:** antiseizure medication, epilepsy, pregnancy, therapeutic drug monitoring

## Abstract

**Objective:**

Information to women with epilepsy on pregnancy‐related antiseizure medication (ASM) issues and reliable tools for therapeutic drug monitoring (TDM) are important aspects of epilepsy care. We aimed to develop and test an online tool for patient education on pregnancy‐related issues and communication with epilepsy nurses during pregnancy for women with epilepsy.

**Methods:**

An existing national platform for online communication (1177.se) was used, and an online tool was developed by two epilepsy nurses, two neurologists, and an IT technician. The tool was launched as a complement to standard care, and patients deciding to use it were invited to participate in a survey of user experiences and knowledge questions.

**Results:**

The online tool consists of two modules: one for patient education and one for TDM during pregnancy. The latter module allows scheduling of automatic reminders of blood tests that are sent to patients at set intervals. The epilepsy nurse can communicate results and suggested dose changes in the tool. A total of 48 women answered the survey: 28 had been invited to use the information module and 20 to use the TDM module. Patient experiences were generally good, and most users of the TDM module would prefer an online means of communication in future pregnancies. For epilepsy nurses, the tool provided good overview of patients currently pregnant and administrative advantages compared with traditional means of communication.

**Significance:**

Online patient education and communication about TDM during pregnancy are feasible and can be a valuable part of future digitalization efforts in epilepsy care.

**Key points**

- We created an online tool for pregnancy‐related information and TDM in pregnancy
- The tool allows automatic reminders of blood tests and communication about dose changes
- A questionnaire was distributed one month after invitation to the information module and one month after delivery for the TDM module.
- A total of 28 responses were collected for the information module and 20 for the TDM module
- Patient satisfaction was high, and the majority would prefer online communication in future pregnancies

## 1 INTRODUCTION

Information to women with epilepsy on pregnancy‐related antiseizure medication (ASM) issues is a core task for epilepsy services and among the American Academy of Neurology epilepsy quality parameters.^1^ Nonetheless, knowledge on pregnancy‐related issues has been found to be inadequate in several studies and more patient education is needed.^2^, ^3^ Women using ASMs with pregnancy‐related changes in pharmacokinetics need to be in frequent communication with epilepsy services for drug‐level monitoring and dose adjustment.^4^

The rapid digitalization of health care facilitates communication, and digital tools are increasingly used in epilepsy care. Examples include self‐management programs in epilepsy, such as the Program of Active Consumer Engagement in Self‐management in epilepsy and Personalized Internet‐Assisted Underserved Self‐management of Epilepsy, which feature digital patient education tools or remote delivery of epilepsy care.^5^, ^6^ Physical meetings with a neurologist have been reported to be the preferred method of receiving information on pregnancy‐related issues in epilepsy,^2^ but Internet‐based solutions can serve as a complement and make patient education material available for revision whenever needed. Mobile device apps have also been used to study ASM compliance during pregnancy.^7^ The COVID‐19 pandemic has further illustrated the importance of tele‐neurology. To our knowledge, no reports exist on digital tools for facilitation of information to women with epilepsy and communication including ASM dose adjustments during pregnancy.

We developed an online tool for information on pregnancy‐related issues for women with epilepsy, and for communication regarding therapeutic drug monitoring (TDM) and dose adjustments during pregnancy. The tool was designed to reduce the need for telephone contacts between epilepsy nurses and patients, a system which often requires many attempted calls before contact is established. Furthermore, we aimed to enhance safety by automatic reminders of TDM and to provide patient education. We here report the design and specifications of the online tool and results from a quality assurance project on patient experiences.

## 2 METHODS

### 2.1 Online tool

**FIGURE 1:**
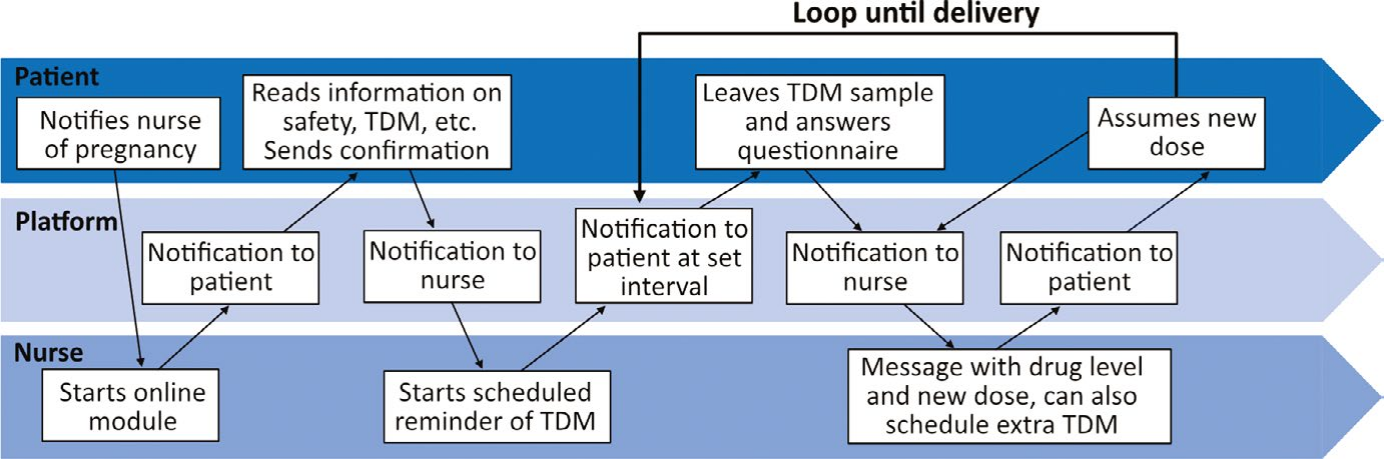
The online tool. Schematic description of the TDM tool and communication between patient and epilepsy nurse

The Swedish national online Patient Portal (www.1177.se) was used.^8^ The platform allows confidential communication and secure identification, and is approved for healthcare use. Two epilepsy nurses (EG and KL) and two neurologists (AET and JZ) developed the modules of the online tool: one containing information for all women with ASM‐treated epilepsy of childbearing age and the other specifically for communication during pregnancy. The information module contains information on ASM interactions with contraceptive pills, malformation risks, advice on folic acid supplementation, delivery, safety issues, and breastfeeding. The pregnancy module contains not only information, but also an interactive part in which the epilepsy nurse sets a schedule for TDM, and messages are sent to the patient at set intervals as a reminder for blood tests. Following a test, the tool allows the epilepsy nurse to send a message with dose changes, after which the new dose is confirmed by the patient (Figure 1). There was no specific training for patients on the tool, but they received a generic folder on how to use the patient portal and instructions by the epilepsy nurse as deemed necessary in each case.

### 2.2 Study population

Women of childbearing age with epilepsy seen by epilepsy nurses from October 2019 were offered to participate and get access to the tool, as a supplement to standard care. Women with epilepsy of childbearing age (information module) and pregnant women with epilepsy (pregnancy module) were invited. Recruitment was not consecutive, but opportunistic.

The prespecified protocol stated that two separate analyses should be performed; the first, which is presented in this manuscript, should be performed after at least 20 participants had answered surveys for both modules. The purpose of this early analysis was to assess feasibility, safety, and patient satisfaction. The target was reached by September 30, 2020.

### 2.3 Questionnaire and response rate

A survey was distributed by mail one month after the invitation to the information module and one month after delivery for the pregnancy module. Patients with miscarriage were excluded. The survey asked about patient experience of the Internet‐based tool and questions of patient knowledge on pregnancy‐related issues in epilepsy. The knowledge questions were selected from KOWIE‐I (Knowledge on Women's Issues and Epilepsy),^3^ to give a survey of the information that was given in the Internet‐based modules. The questions were slightly altered/adapted (Supplementary Material) to the content presented in the online tool and translated by one of the authors (JZ).

### 2.4 Safety

Because of the novelty of the tool, it was introduced gradually and monitored for safety incidents by the epilepsy nurses.

### 2.5 Statistical analysis

For questions about the online tool user‐friendliness and relevance, only answers from women that stated that they had accessed the tool were included. SPSS version 25 (IBM) was used for all analyses. Continuous variables are presented with median and range, and categorical variables including survey answers are presented as proportions with 95% confidence intervals.

## 3 RESULTS

### 3.1 Study population

A total of 48 women were sent the survey for the information module and a total of 31 women for the pregnancy module. Responses were received from 28 (58%) and 20 (65%), respectively. The characteristics of the study cohort are given in Table 1.

**TABLE 1:** Study cohort. Age, education level, and epilepsy/obstetric history. Frequencies and proportion represent positive answers to the questions

	Information, n = 28	Pregnancy, n = 20
	Median (min‐max)	Median (min‐max)
Age	28.5 (19‐37)	33 (27‐44)
Epilepsy Duration, years	8 (0‐27), n = 23	14 (2‐38), n = 16

Highest level of education	n (%)	n (%)
Compulsory school (9 y)	4 (14%)	1 (5%)
High school	13 (46%)	6 (30%)
University	11 (39%)	13 (65%)
Seizures during the past 12 mo	13 (46%)	5 (25%)
Have you been pregnant previously?	8 (29%)	
Did you have epilepsy during your last pregnancy?	3 (38%), n = 8	
Do you plan pregnancy in <2 y?	17 (65%), n = 26	
Have you accessed the information /or TDM module on 1177.se?	24 (86%)	20 (100%)

Abbreviation: TDM, therapeutic drug monitoring.

### 3.2 Patient experiences

**FIGURE 2:**
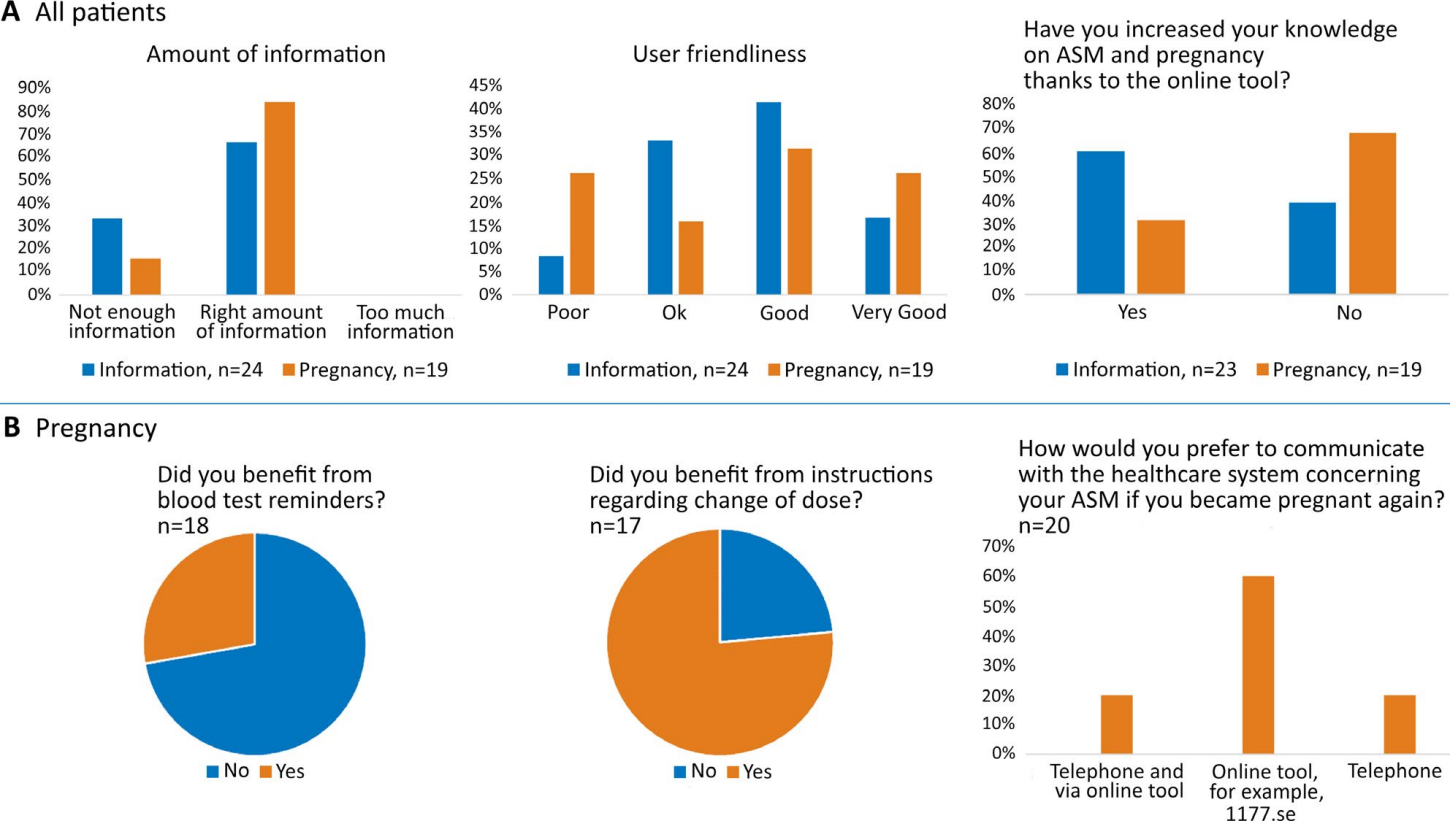
User experiences A, Perceived amount of information, user‐friendliness, and increased knowledge. B, Questions specific for users of the pregnancy module for TDM

For the information module, patient satisfaction was high. Twenty patients (83%, 95% CI: 65‐94) answered that the information given online was a good supplement to the information provided by physicians or nurses. Fourteen patients (61%, 95% CI: 41‐79) answered that their knowledge on pregnancy‐related issues had increased because of the information online. The user‐friendliness of the tool was considered good or very good by a majority of respondents (Figure 2A).

For the pregnancy module, 12 patients (60%, 95% CI: 38‐79) answered that they would prefer to use an online tool again for communication regarding their epilepsy, four patients (20%, 95% CI: 7‐41) would prefer a combination of online communication and telephone, and four patients (20%, 95% CI: 7‐41) preferred exclusively telephone communication. Only six patients (32%, 95% CI: 14‐54) answered that their knowledge on pregnancy‐related issues had increased by reading the online information. Eight patients (47%, 95% CI: 25‐70) answered that the tool was good or very good in terms of user‐friendliness. Reminders about blood tests were perceived useful by five patients (28%, 95% CI: 12‐51), whereas 13 patients (77%, 95% CI: 53‐92) found the communication regarding dose changes useful (Figure 2B).

In free‐text comments, some patients commented that they would have preferred more detailed information and some that they would have liked more freedom regarding the order in which they could access the information. Regarding technical issues, some patients preferred more initial assistance with setting up the tool correctly regarding text message/email reminders.

### 3.3 Safety

Because of the novelty of the tool, it was introduced in parallel with existing practices. During the study period, there was one safety incident, when a patients’ confirmation that a blood sample had been taken was overlooked. As a consequence, no instructions on dose change were given and the patient did not leave any more samples. There were no seizures throughout the pregnancy. Following this incident, we have tried to increase vigilance regarding monitoring of the results of blood tests in the laboratory database, which is independent of the online tool and provides a safety net against similar future incidents.

### 3.4 Patient knowledge

Overall, the patients answered correctly to the knowledge questions (Table 2).

**TABLE 2:** Knowledge questions. Proportions of responders stating that they had accessed the online information. The questionnaire was in Swedish

	Information n = 24		Pregnancy n = 17‐18	
	n	% (95% CI)	n	% (95% CI)
Can folic acid before and during the first trimester reduce birth defect risks linked to ASM treatment?				
Yes	13	54 (35‐73)	16	94 (76‐99)
Maybe	4	17 (6‐35)	0	
No	2	8 (2‐24)	1	6 (1‐24)
Don't know	5	21 (8‐40)	0	
Do all ASM carry the exact same birth defect risk?				
Yes	0	0	0	0
No	24	100	17	100
Can babies be affected by ASM during pregnancy or through breastfeeding?				
Yes	20	83 (65‐94)	14	82 (60‐95)
No	2	8 (2‐24)	3	18 (5‐40)
Don't know	2	8 (2‐24)	0	0
Can pregnancy alter the metabolism of ASM so that doses need to be increased to stop seizures?				
Yes	24	100	16	89 (69‐98)
No	0	0	0	0
Don't know	0	0	2	11 (2‐31)
How long should a woman be seizure‐free before pregnancy for a low likelihood of seizures during pregnancy?				
3 mo	1	4 (0.5‐18)	0	0
9 mo	0	0	1	6 (1‐23)
12 mo	4	17 (6‐35)	2	11 (2‐31)
2 y	2	8 (2‐24)	1	6 (1‐23)
Don't know	17	71 (51‐86)	14	78 (55‐92)

## 4 DISCUSSION

We here describe the development and implementation of an online tool for enhanced patient education and improved communication during pregnancy for women with epilepsy. The main finding is that implementation is feasible and patient satisfaction with the tool is positive. There seems to be a high acceptance of online information on pregnancy‐related issues in women with epilepsy of childbearing age in Sweden, and also willingness to use Internet‐based communication regarding TDM and dose adjustments. Our main conclusion is therefore that online communication regarding ASM and epilepsy can be useful in epilepsy care, at least in countries with a fair level of digitalization. A prerequisite for the development of our tool was the availability of an existing online platform for secure communication between patients and healthcare providers.

Our findings are well in line with previous work on digitization of health care. Mobile device apps have been used to study ASM compliance during pregnancy.^7^ The COVID‐19 pandemic has also led to the rapid development of online communication tools for epilepsy caregivers,^9^ and as patients become more used to online care, demand for online TDM solutions during pregnancy is likely to increase.

Our study adds some specific knowledge on what should be included in an online tool for patient education on reproductive aspects, and TDM during pregnancy. Most pregnant women appreciated receiving instructions about dose adjustments online, which seems to be a key feature. Some patients would have preferred more specific training, and the tool should ideally be very easy to navigate. The knowledge questions included in the survey indicate that the respondents were generally well‐informed regarding ASM treatment and pregnancy. Users of the pregnancy module did not feel that their knowledge increased much, which is satisfactory given that such information is supposed to be given well before pregnancy. We did not have enough responders that had been invited but not used the tool for analysis of the contribution of our tool to the patient knowledge level, but hope to return to that issue in subsequent studies.

The experiences of the epilepsy team (physicians and nurses) of the online tool were generally positive. The main advantages were that administration became much easier; the system provides an overview of all women that are currently pregnant, and the automatic scheduling makes it easy to track when blood tests are expected. Other advantages include that nurses no longer need to reach patients by phone to convey messages of dose changes and that patients have an easy means of contacting the epilepsy team. At the time of the research, 98 women had used or were using the tool.

There are limitations to our study and the representativeness of our findings. The number of respondents is relatively low, but still represents data collection for more than a year. Sweden is a country where Internet access is high. Many inhabitants are used to the online platform 1177.se for communication with healthcare providers, and transition to the tool for women with epilepsy may have been easier than if the system would have needed to be developed entirely de novo. Not all patients that were invited to the information module actually accessed the tool, so Internet‐based information can hardly replace traditional patient education in clinic. The response rate was relatively high, but nonresponders may have been more dissatisfied than responders.

This study is the first of a two‐level evaluation. Because of the novelty of the tool, we wanted to perform an early evaluation determining feasibility, safety, and patient experiences. We also plan a second evaluation after one hundred users have responded to the survey; the scale of that analysis will hopefully allow subgroup analyses for the identification of ideal target groups for the tool and differences in knowledge between users and nonusers.

We conclude that online resources may improve counseling of women with epilepsy on pregnancy‐related issues. Online tools can be a valuable part of the toolbox in future tele‐neurology developments in epilepsy.

## CONFLICT OF INTEREST

Outside of the submitted work, JZ has been a speaker at nonbranded educations organized by UCB and Eisai and as an employee of Sahlgrenska University Hospital he has been or is an investigator in clinical trials sponsored by GW Pharma, Bial, UCB, and SK Life Science (no personal compensation). No other authors have any competing interest to disclose. We confirm that we have read the Journal's position on issues involved in ethical publication and affirm that this report is consistent with those guidelines.

## ETHICAL APPROVAL

The study was approved by the Ethics Review Authority (No. 2019‐02630), the relevant government agency. All patients provided written informed consent.

## Supporting information

Supplementary Material

## Data Availability

The data of the study are protected by confidentiality laws and cannot be shared by the authors.
