# Supplementary material for: An online tool for information to women with epilepsy and therapeutic drug monitoring in pregnancy: Design and pilot study
Source: Epilepsia Open. 2021 Feb 20;6(2):339–44. doi: 10.1002/epi4.12473 (PMC8166785; doi:10.1002/epi4.12473)
Supplement: Supplementary file 1 — Supplementary Material [file EPI4-6-339-s001.docx]

## Questions asked in the survey on communication via 1177.se regarding epilepsy medication during pregnancy

What is your highest level of education? Primary school high school university

How long have you had epilepsy (in years)?:

Have you had seizures during the past 12 months: Yes No

Which antiseizure medications do you take and in which doses?

| Drug name | Doses |
| --- | --- |
|  |  |
|  |  |
|  |  |

How many times have you been pregnant? 1 2 3 more

Have you had seizures during your last pregnancy? Yes No

Was the dose of your medications adjusted during your last pregnancy? Yes No

How long ago was your pregnancy (number of months since the termination of the pregnancy)?:

### Questions regarding the epilepsy service on 1177.se

Did you use 1177.se to receive information during your last pregnancy? Yes No

What is your opinion on

The amount of information: not enough information right amount too much information

User friendliness: bad ok good very good

Did you use 1177.se to receive test results and adjust doses during your last pregnancy? Yes No

Did you benefit from:

Blood test reminders Yes No

Dose adjustment instructions Yes No

What is your opinion on the user friendliness: bad ok good very good

How would you prefer to communicate with health care services regarding your epilepsy medication if you were to become pregnant again?:

By telephone Via online service, such as 1177.se

Do you think that you have increased your knowledge regarding epilepsy medication and pregnancy by reading the information 1177.se? Yes No

May we contact you for an interview if we have further questions? Yes No

You are welcome to write down your comments regarding your experience on the back of this page.

# Knowledge questions

Below are some questions regarding antiepileptic drugs and pregnancy. Answer directly without looking up the answers on the internet or in other ways. Answer by circling the correct option.

1. Can folic acid supplements before and during the first part of pregnancy reduce the risk of malformation during treatment with antiepileptic drugs?

Yes Maybe No Don’t know

1. Can children be affected by antiepileptic drugs during the time in the womb or through breast feeding?

Yes No Don’t know

1. Can pregnancy change the way the body takes care of (breaks down) antiepileptic drugs so that the dose needs to be adjusted in order to prevent seizures?

Yes No Don’t know

1. Preferably for how long should a woman be seizure free prior to a pregnancy, in order to have a low probability for seizures during the pregnancy?

3 months 9 months 12 months 2 years Don’t know

1. Do all antiepileptic drugs give exactly the same risk of malformations?

Yes No

(If you have any questions regarding the correct answers, you may check them afterwards via the online service. You are also welcome to call the epilepsy nurse.)
